# Pituicytoma

**DOI:** 10.4103/2152-7806.73802

**Published:** 2010-12-13

**Authors:** Rafael Augusto Castro Santiago Brandão, Moises Heleno Vieira Braga, Atos Alves de Souza, Baltazar Leão Reis, Franklin Bernardes Faraj de Lima

**Affiliations:** Department of Neurosurgery, Santa Casa Hospital, Faculty of Medical Science of Minas Gerais, Belo Horizonte, Brazil

**Keywords:** Glioma, hypophysis, neurohypophyseal tumor, pituicytes, pituicytoma, pituitary gland, pituitary tumors

## Abstract

**Background:**

Pituicytomas originate from pituicytes, modified glial cells derived from ependymal lineage that are found in the stalk and posterior lobe of pituitary gland. The clinical presentation is similar to other pituitary tumors and imaging exams may suggest pituitary adenoma. The diagnostic is based on histopathological analysis. Surgical treatment can be performed by transsphenoidal approach with good results. The prognostic is good after total tumor resection.

**Case Description:**

We describe here the case of a 17-year-old patient with a history of persistent headache and visual disturbances. Magnetic resonance imaging demonstrated an enhancing solid sellar mass suggestive of pituitary adenoma. The intrasellar mass was resected through a transsphenoidal approach and the diagnosis of pituicytoma was made after histopathological analysis.

**Conclusion:**

Pituicytomas are rare tumors of the neurohypophysis derived from pituicytes. Their clinical presentation resembles that of non-functional pituitary adenomas, but these two types of tumors are histologically well distinct.

## INTRODUCTION

Pituicytomas are very rare primary tumors of the neurohypophysis and can affect both the sellar and suprasellar regions. Few cases have been described in the literature.[17] Until now there are 31 cases reported, all of them are described in Table 1, which contribute to the poor characterization of the tumor and consequent diagnostic difficulties.

**Table 1:** Summary of the reported 31 cases of pituicytoma

Patient no.	Series (ref. no.)	Age (year)/sex	Presentation	Imaging	Resection	Follow up	Recurrence/complications	Radiation therapy
1	Hurley *et al*., 1994 (7)	26/F	Decreased visual acuity and hemianopsia	2-cm enhancing sellar mass, T1-isointense, T2- hyperintense	TSP/STR	3 year	None/transient Deficit	5040 cGy
2	Brat *et al*., 2000 (2)	55/F	Visual deficit	Suprasellar, enhancing	GTR	1 year	None	None
3	Brat *et al*., 2000 (2)	30/M	Headache	Suprasellar, enhancing	GTR	1 year	None	None
4	Brat *et al*., 2000 (2)	39/M	Headache	Solid and cystic, intrasellar, enhancing	TSP/GTR	2 year	None	None
5	Brat *et al*., 2000 (2)	42/M	Hypopituitarism/hemianopsia	Intrasellar, enlarged over 2 years of observation	TSP/STR	2.5 year	2 year progression with resection	None
6	Brat *et al*., 2000 (2)	42/M	Visual deficit/decreased libido	Solid, suprasellar enhancing	STRl	1 year	Re-resection for recurrence × 2 at 5 and 15 months	None
7	Brat *et al*., 2000 (2)	46/M	Hypopituitarism	Solid, suprasellar enhancing	GTR	8 year	None	None
8	Brat *et al*., 2000 (2)	83/F	Visual deficit	Suprasellar	GTR	2 year	None	None
9	Brat *et al*., 2000 (2)	48/M	Hypogonadism	2-cm solid, suprasellar mass encasing vessels	Craniotomy/STR	8 months	Recurrence at 5 months with subtotal re-resection	None
10	Brat *et al*., 2000 (2)	51/F	Visual deficit	Solid, enhancing sellar mass consistent with adenoma	GTR		Unknown	None
11	Schultz *et al*.,2001 (16)	66/M	Decreased visual acuity, visual field deficit	2-cm enhancing, T1- isointense, T2-hyperintense	TSP/GTR	2 year	None	None
12	Cenacchi *et al*., 2001 (3)	79/F	Hypopituitarism/visual disturbances	Unknown	TSP/GTR	6 months	None	None
13	Figarella- Branger *et al*., 2002 (5)	59/M	Hypopituitarism	Solid, enhancing	TSP/STR	11 year	None	None
14	Figarella- Branger *et al*., 2002 (5)	46/M	Decreased libido/hypogonadism	Solid, enhancing, suprasellar	GTR	4 year	None/transient hemiparesis	None
15	Figarella- Branger *et al*., 2002 (5)	58/M	Hypopituitarism/memory deficits	Solid, enhancing, mimicking posterior clinoid meningioma	GTR	2 year	None/DI	None
16	Uesaka *et al*., 2002 (20)	34/M	Decreased visual acuity	Solid, enhancing, T1- isointense, T2-hyperintense	TSP/STR	3 months	None	None
17	Katsuta *et al*., 2003 (8)	32/F	Amenorrhea/visual field defect	Intrasellar, isointense T1 and T2, enhancing	TSP/GTR	2 year	None/DI	None
18	Ulm *et al*., 2004 (21)	45/M	Decreased libido/low testosterone	2-cm solid, enhancing, suprasellar	Craniotomy/STR	Unknown	None	Stereotactic radiation
19	Kowalski *et al*., 2004 (9)	52/M	Panhypopituitarism	Solid, heterogeneously enhancing sellar/suprasellar mass	TSP/STR	11 months	Recurrence	Fractionated radiation after recurrence
20	Shah *et al*., 2005 (17)	32/F	Amenorrhea/headache	Heterogeneously enhancing posterior pituitary mass, T1- isointense, T2-hyperintense	TSP/STR	5 year	Recurrence with re-resection TSP	None
21	Shah *et al*., 2005 (17)	45/F	Headache	Enhancing sellar/ suprasellar mass, T1- isointense, T2-hypointense	TSP	Unknown	Unknown	Unknown
22	Chen, 2005 (4)	54/M	Headache	Enhancing sellar/suprasellar mass	TSP/STR	16 months	None	None
23	Takei *et al*.,2005 (18)	54/F	Incidental at autopsy	None	None	None	None	None
24	Nakasu *et al*., 2006 (12)	42/F	Amenorrhea	Homogeneously enhancing sellar/suprasellar mass	Craniotomy/STR	5 year	None	None
25	Nakasu *et al*., 2006 (12)	62/F	Headache/fatigue	Homogeneously enhancing sellar/suprasellar mass	Craniotomy/STR	1.5 year	None/transient DI/hypopituitarism	None
26	Benveniste *et al*., 2006 (1)	47/M	Hemorrhage/low LH/FSH	Hemorrhagic suprasellar mass with IVH	Craniotomy/STR	None	Unknown	None
27	Gibbs *et al*., 2006 (6)	64/M	Bitemporal hemianopsia	Homogeneously enhancing 3-cm suprasellar mass, T1- isointense, T2-hyperintense, angiogram hypervascular capillary blush from ICA only	Cranio-orbitozygomatic craniotomy/GTR, very vascular	Unknown	Unknown	Unknown
28	Thiryayi at al, 2007 (19)	77/M	Hypogonadism Quadrantonopia bitemporal inferiorSuprasselar level	TSP/ STR	TSP/ STR	None	None	None
29	Wolfe *et al*.2008 (22)	71/F	Decreased visual acuity and visual field defects	Solid, enhancing mass	TSP/STR	1.5 year	None	None
30	Orrego J. T. 2009(14)	55/M	Decreased libido Dysfunction erectic ginecomastia	Suprasella mass isointense on T1	TSP/STR	None	None	None
31	Brandao and Braga *et al*. 2010	17/M	Headache visual Disturbance	Contrast-enhance solid sellar	TSP/STR	24 months	None	None

TSP: Transphenoidal Approach, STR: subtotal resection, GTR: gross-total resection

Pituicytomas originate from pituicytes, modified glial cells derived from ependymal lineage which are found in the stalk and posterior lobe of pituitary gland.[13,15] On neuroimaging, pituicytomas are solid, uniformly contrast-enhancing masses. They are histologically low grade, featuring only mild nuclear atypia and no mitotic activity.[2] We describe here a case of pituicytoma diagnosed at our service and discuss it in relation to the data available in the literature.

## CASE REPORT

### Description

**Figure 1:**
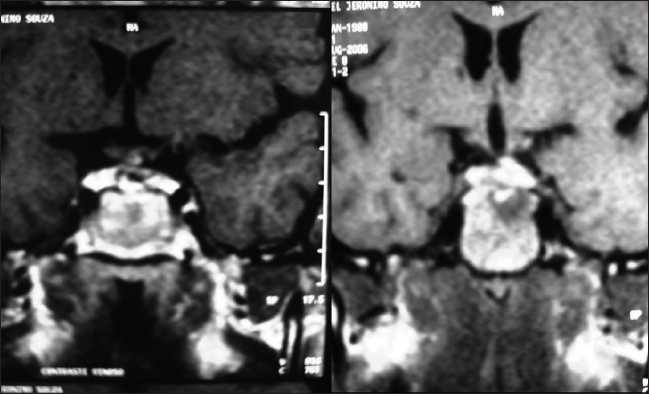
Preoperative MRI.

**Clinical presentation:** A 17-year-old boy was reported with a history of persistent headache and recent onset of visual disturbances. Examination revealed bitemporal heteronymous hemianopsia and swelling of the left optic papilla. The remaining neurological exam was normal. Magnetic resonance imaging (MRI) of the brain revealed a contrast-enhancing, expansive solid sellar and suprasellar mass with an intermediate signal intensity on T1- and T2-weighted images, measuring about 2.6 cm × 1.6 cm × 1.5 cm [Figure 1]. The mass occupied the sella turcica, extending from the suprasellar cisterna and compressing optic chiasm and chiasmatic recess of the third ventricle. Endocrinological analysis demonstrated mild hyperprolactinemia (31.75 ng/ml, reference: 2.1–17.7 ng/ml), with the other pituitary hormones being normal.

**Surgery:** The patient was submitted to partial transphenoidal resection of the tumor. The surgical aspect did not differ from that found in a pituitary adenoma, both in consistency, color, as well as in bleeding. The cavity was filled with autologous fat.

**Postoperative period:** The patient presented no major complications, except for diabetes insipidus detected during the early postoperative period which was completely controlled. Residual tumor was identified, but no tumor recurrence was observed after a follow-up period of 24 months.

**Figure 2:**
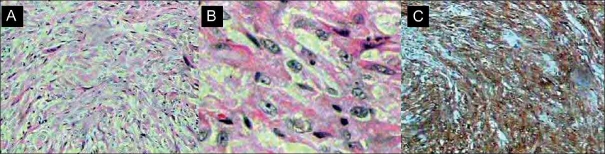
(A) Microscopic view showing a fusocellular tumor consisting of pleomorphic cells with a fascicular growth pattern. (B) Cells with eosinophilic citoplasm and clearly visible nucleoli. (C) Immunohistochemical reaction with S-100 protein.

**Pathological anatomy:** Microscopy showed a predominantly fusocellular tumor consisting of pleomorphic cells with a fascicular growth pattern. The cells were characterized by abundant eosinophilic cytoplasm, vesiculous nucleo with mild atipia and clearly visible nucleoli. Mitotic figures were occasionally observed [Figure 2].

The material was analyzed by immunohistochemical study. Antibodies against several antigens were tested and are shown in Table 2. Most tumor cells were reactive to the cell proliferation antigen Ki-67 and to protein S-100. There was no reaction to the other antigens tested, including glial fibrillary acidic protein (GFAP). According to the criteria proposed by Brat *et al*.,[4] the diagnosis of pituicytoma was thus confirmed.

**Table 2:** Antibodies tested in immunohistochemical study

Antibodies	Clone	Results
Proliferation antigen Ki-67	MIB-1	Positive
Epithelial membrane antigen – EMA	E29	Negative
Cytokeratins of 40, 48, 50, and 50.6 kDa	AE1/AE3	Negative
Protein S-100	Policlonal	Positive
Glial fibrillary acidic protein – GFAP	Policlonal	Negative
Synaptophysin	Sy38 AFP	Negative
CD68 – lysosomal protein	KP1	Negative
Nerve growth factor receptor	NGFR5	Negative
Melanoma	PNL2	Negative
Melanoma-associated gp100 antigen	HMB-45	Negative
Melanoma antigen recognized by T cells – Melan A/MART-1	A103	Negative

## DISCUSSION

The neurohypophysis comprises the posterior portion of the pituitary, infundibulum, and tuber cinerium.[7,17] The cellular elements that form the posterior part of the pituitary include microglia, pituicytes, and terminal axons of secretory neurons of the hypothalamus. Pituicytes are modified microglial cells that occupy perivascular areas of the neurohypophysis and participate in the regulation of the release of hypothalamic hormones. The cells are spindle shaped and normally react to GFAP.[2,6] Five types of pituicytes have been described based on the histopathological criteria of Takei *et al*.,[7,10,18] (1) major pituicytes, the most common type characterized by an oval or irregular nucleus, distinct nucleoli, and cytoplasm containing various organelles; (2) dark pituicytes which present the same structure as major pituicytes, but have an electron-dense cytoplasm; (3) oncocytic pituicytes characterized by a large number of mitochondria; (4) ependymal pituicytes which are rudimentary ependymal cells; and (5) granular pituicytes which contain numerous electron-dense granules and give origin to granular cell tumors or choristomas.

The most common pituitary tumors are adenomas originating from the adenohypophysis. Although rare, posterior pituitary tumors include hamartomas, craniopharyngiomas, germinomas, granular cell tumors, meningiomas, pituicytomas, and pilocytic astrocytomas.[2,7]

Few cases of primary tumors of the neurohypophysis have been described, a fact impairing the classification of these tumors. So far, 30 cases of pituicytomas have been published [Table 1].[1,3,5,9,11,12,14,16,19–22] In 2000, Brat *et al*.,[2] described nine cases of pituicytomas and provided a more precise and detailed characterization of the clinical and pathological findings related to this tumor. In addition, the authors proposed a clearer and more objective definition of the tumor.[6,8]

Pituicytomas are rare, noninfiltrative tumors of glial origin, which arise in the neurohypophysis that comprises the posterior region of the pituitary and the pituitary stalk.[2] In the past, the term pituicytoma frequently included granular cell tumors or choristomas and pilocytic astrocytomas.[6] Today, this term is reserved for low-grade glial tumors classified as grade I by the World Health Organization and differing from astrocytomas.[11] Thus, as in the present case, pituicytomas are solid well-defined tumors, which appear isointense on T1-weighted MRI and are characterized by marked vascular proliferation and intense contrast enhancement. These findings are nonspecific and confirmation by anatomopathological analysis is necessary.[6,11]

Histologically, pituicytomas are characterized by spindle-shaped cells arranged in interlacing fascicles. Nuclei is oval to elongated with a mild irregularity. The citoplasm is eosinophilic and homogeneous which presents little or no granulation or vacuolization and necrosis is absent.[2] Pituicytomas are histologically classified as low-grade gliomas which present little nuclear atypia and rare mitotic activity. Most tumor cells are reactive to the cell proliferation antigen Ki-67 and to protein S-100. The cells normally react to GFAP and present little or no cytoplasmic reactivity to epithelial membrane antigen (EMA). In the series described by Brat *et al*.,[2] one of the nine cases showed no reactivity to GFAP and wide variation in the intensity of the reaction was observed in the remaining eight positive cases. In the present patient, the tumor consisted of spindle-shaped cells with a fascicular organization and abundant granular cytoplasm and showed intense positivity to the Ki-67 and protein S-100 antigens, findings compatible with pituicytoma, and no reactivity to EMA or GFAP.

The most common clinical presentation was headache and bitemporal hemianopsia due to compression of the optic chiasm.[11] Other signs frequently observed are pan-hypopituitarism and mild hyperprolactinemia.[2] The patient may even be completely asymptomatic, and the tumor is detected incidentally. The present patient had mild hyperprolactinemia, with the other pituitary hormones being normal, associated with persistent headache and bitemporal hemianopia. These findings, together with the imaging detection of a solid and well-delimited mass in the sellar region, may lead to the clinical diagnosis of pituitary adenoma. The indicated treatment is surgery which can be performed by the transsphenoidal approach. The present patient underwent transsphenoidal resection of the tumor. Tumor consistency and increased bleeding observed during surgery are essential data that help the pathologist with the diagnosis of the tumor. Some authors have reported a greater bleeding tendency of these tumors during surgery.[7] In the present case, no perioperative alterations that would distinguish the tumor from a macroadenoma were observed and the diagnosis was exclusively made by histological analysis.

No tumor recurrence was observed in the related case after a follow-up period of 24 months. Although few studies are available in the literature, data suggest a high rate of tumor recurrence after partial resection and a good prognosis, with little or no recurrence, after total resection.[4]

## CONCLUSION

Pituicytomas are rare tumors of the neurohypophysis derived from pituicytes. Their clinical presentation resembles that of non-functional pituitary adenomas, but these two types of tumors are histologically well distinct. Surgery is the indicated treatment with a good prognosis if the tumor is completely resected.
